# D181A Site-Mutagenesis Enhances Both the Hydrolyzing and Transfructosylating Activities of BmSUC1, a Novel β-Fructofuranosidase in the Silkworm *Bombyx mori*

**DOI:** 10.3390/ijms19030683

**Published:** 2018-02-28

**Authors:** Quan Gan, Xin Li, Xinwei Zhang, Lanlan Wu, Chongjun Ye, Ying Wang, Junshan Gao, Yan Meng

**Affiliations:** 1 School of Life Sciences, Anhui Agricultural University, Hefei 230036, China; gq517988@163.com (Q.G.); guaguaallen@hotmail.com (X.L.); wangdeng4242@sina.com (X.Z.); wulanlan1208@163.com (L.W.); yechj1983@163.com (C.Y.); wang1994ying@126.com (Y.W.); gaojsh@ahau.edu.cn (J.G.); 2 Anhui International Joint Research and Development Center of Sericulture Resources Utilization, 130 West Changjiang Road, Hefei 230036, China

**Keywords:** *Bombyx mori*, β-fructofuranosidase, site-directed mutagenesis, hydrolysis, transfructosylation

## Abstract

β-fructofuranosidase (β-FFase) belongs to the glycosyl-hydrolase family 32 (GH32), which can catalyze both the release of β-fructose from β-d-fructofuranoside substrates to hydrolyze sucrose and the synthesis of short-chain fructooligosaccharide (FOS). *BmSuc1* has been cloned and identified from the silkworm *Bombyx mori* as a first animal type of β-FFase encoding gene. It was hypothesized that BmSUC1 plays an important role in the silkworm-mulberry adaptation system. However, there is little information about the enzymatic core sites of BmSUC1. In this study, we mutated three amino acid residues (D63, D181, and E234) that represent important conserved motifs for β-FFase activity in GH32 to alanine respectively by using site-directed mutagenesis. Recombinant proteins of three mutants and wild type BmSUC1 were obtained by using a Bac-to-Bac/BmNPV expression system and BmN cells. Enzymatic activity, kinetic properties, and substrate specificity of the four proteins were analyzed. High Performance Liquid Chromatography (HPLC) was used to compare the hydrolyzing and transfructosylating activities between D181A and wtBmSUC1. Our results revealed that the D63A and E234A mutations lost activity, suggesting that D63 and E234 are key amino acid residues for BmSUC1 to function as an enzyme. The D181A mutation significantly enhanced both hydrolyzing and transfructosylating activities of BmSUC1, indicating that D181 may not be directly involved in catalyzation. The results provide insight into the chemical catalyzation mechanism of BmSUC1 in *B. mori*. Up-regulated transfructosylating activity of BmSUC1 could provide new ideas for using *B. mori* β-FFase to produce functional FOS.

## 1. Introduction

Invertase or β-fructofuranosidase (β-FFase) (EC 3.2. 1.26) catalyzes the release of β-fructose from the nonreducing termini of various of β-d-fructofurnoaside substrates [1], such as sucrose, raffinose, inulin, and levan [2]. In addition to hydrolyzing sucrose, β-FFase may also catalyze the synthesis of short-chain fructooligosaccharides (FOS), in which one to three fructosyl moieties are linked to the sucrose skeleton by different glycosidic bonds, depending on the enzyme source [3]. The reaction proceeds by a double displacement mechanism in which a covalent glycosyl-enzyme intermediate is formed [4]. The breakdown of sucrose is widely used as a carbon or energy source by bacteria, fungi, and plants. In plants, both glucose and fructose are implicated in the signaling pathways by which sucrose concentration functions as a key sensor of the nutritional status of plants. Thus, invertase plays a fundamental role in controlling cell differentiation and development [5,6]. Bacterial fructosyltransferases catalyze two different reactions, depending on the nature of the acceptor. This results in transglycosylation when a fructan chain is used as an acceptor or in hydrolysis, when water is used as the acceptor [7]. Fructans are one of the most promising ingredients for functional food and non-food industries and have prebiotic properties. They exert a beneficial effect on human health through improving calcium and magnesium absorption in adolescents and in postmenopausal women [8], and to help prevent cardiovascular diseases, colon cancer, or osteoporosis [9,10,11,12].

β-FFases are common in microorganisms and plants and their catalyzing properties and protein crystal structures are well studied. However, β-FFase is rare in the animal kingdom although its activity has been found in several Lepidoptera insects [13,14,15]. A β-FFase gene was cloned and identified from the silkworm *Bombyx mori* and termed *BmSuc1*. This provided the first direct evidence for the existence of β-FFase in an animal genome [16]. In *B. mori*, BmSUC1 may play a role in avoiding the toxic effects of the sugar-mimic alkaloids 1,4-dideoxy-1,4-imino-d-arabinitol (D-AB1), and 1-deoxynojirimycin (DNJ) that are present at high concentrations in mulberry latex [16,17]. β-FFase genes have now been found in the larvae of *Helicoverpa armigera* [18], *Manduca sexta* [19], and two Coleopterans *Sphenophorus levis* [20], and *Agrilus planipennis* [21]. In *A. planipennis* and *S. levis*, the insect β-FFase genes are thought to have been acquired through horizontal gene transfer (HGT) from bacteria [20,21], and this supports previous propositions, including the origin of the *B. mori* gene *BmSuc1* [15,16,18,19,22]. There is scant knowledge about the enzymatic activity center and the catalyzing kinetics of insect β-FFases.

β-FFase are classified as family 32 of the glycosyl-hydrolase (GH32). Three conserved motifs in GH32, each containing a key acidic residue, Asn-Asp-Pro-Asn-Gly (NDPNG), Arg-Asp-Pro (RDP) and Glu-Cys (EC) have been implicated in substrate binding and hydrolysis [23]. In this study, we performed site-directed mutagenesis to probe the roles of the three well conserved acidic amino acid residues, Asp63, Asp181, and Glu234 in BmSUC1. The recombinant proteins of wtBmSUC1 and the three mutants were expressed using a Bac-to-Bac/BmNPV expression system [24] and enzymatic assays were conducted to analyze and compare their enzyme characteristics. Our data revealed that the D63A and E234A mutations lost activity, and the D181A mutation significantly enhanced both hydrolyzing and transfructosylating activities of BmSUC1. The results provide insight into the chemical catalyzation mechanism of BmSUC1 in *B. mori* and help to better understand the function of such novel insect β-FFases.

## 2. Results

### 2.1. Analysis of Homology and Conserved Motifs

**Figure 1:**
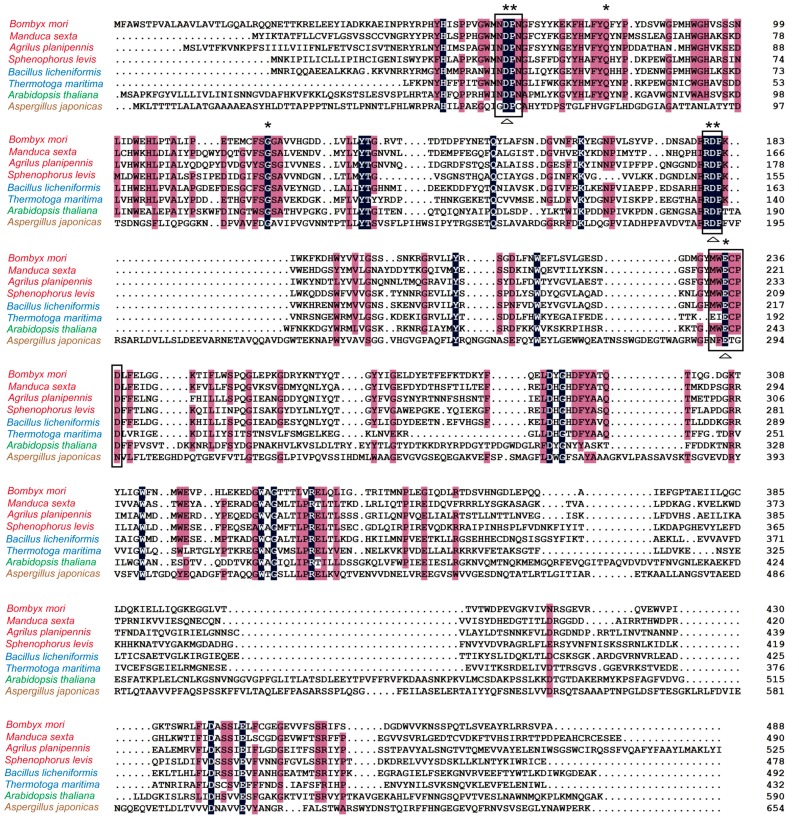
Multiple sequence alignments of β-FFase from *Bombyx mori* (NP_001119721), *Manduca sexta* (ACX49762), *Agrilus planipennis* (AIR93898), *Sphenophorus levis* (AIL92341), *Bacillus licheniformis* (WP_003186470), *Thermotoga maritima* (PDB: 1UYP_A), *Arabidopsis thaliana* (AAA63802) and *Aspergillus japonicus* (ADK46938). The letters with black background are 100% identical among the sequences. The characters with pink background show >75% identity among the sequences. Asterisks indicate the putative substrate-binding sites. The three conserved motifs are boxed, in which each putative catalytic residue is indicated by a triangle. The organism names are presented with different colors (red for insect, blue for bacterium, green for plant and brown for fungus).

The coding region of *BmSuc1* was 1464 base pairs and encoded a 488-amino acid polypeptide with a molecular mass of about 56 kDa [16]. Alignment of multiple amino acid sequences of β-FFases showed that the identity was highest (46%) between BmSUC1 and *Bacillus licheniformis* and was about 40% between BmSUC1 and other insect species (Table 1). We found that five putative substrate binding sites in the GH32 family [23] are well conserved in BmSUC1 and other species including insects, bacteria, plants and fungi (Figure 1). Three motifs Asn-Asp-Pro-Asn-Gly (NDPNG), Arg-Asp-Pro (RDP), and Glu-Cys (EC), each containing a predicted catalytic residue, are completely present in BmSUC1 at positions of Asp63, Asp181, and Glu234 (Figure 1). 

### 2.2. Site Mutagenesis of BmSUC1 and In Vitro Expression of the Recombinant Proteins

**Figure 2:**
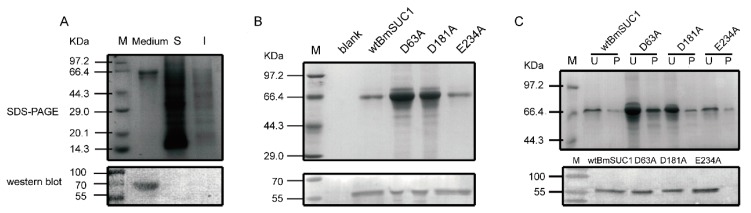
Identification and purification of the enzymes expressed in BmN cells by SDS-PAGE (upper) and western blot (lower) analysis using anti-BmSUC1 antibody. (**A**) wtBmSUC1 expression in the medium and cells (S, supernatant, I, insoluble component); (**B**) Expression of wtBmSUC1 and three mutants in the medium. Blank, cell culture medium after empty vector infection; (**C**) unpurified (U) and purified (P) proteins from the medium. 0.5 μg of each purified protein was identified by western blot.

To analyze the importance of Asp63, Asp181, and Glu234 for BmSUC1 activity, we performed site-directed mutagenesis by PCR. The three residues were artificially mutated to alanine and named D63A, D181A and E234A, respectively (Table 2, Figure S1A). Also, wild type BmSUC1 (wtBmSUC1) was expressed and purified in a bacterial expression system and anti-BmSUC1 polyclonal antibody was prepared. Subsequently, wtBmSUC1 and the mutants were co-expressed with GFP in BmN cells using a Bac-to-Bac/BmNPV expression system (Figure S1B). After two episodes of multiplication from P0 to P2, the virus titers appeared to be increased based on an increase of green fluorescence. This indicated that abundant recombinant proteins were expressed 72 h after transfection by P2 virus (Figure S2B). Through the Sodium dodecyl sulfate-polyacrylamide gel electrophoresis (SDS-PAGE) and immunoblot analysis, we found that BmSUC1 was present in the culture medium, rather than intracellular (Figure 2A). Therefore, the large expression of the four recombinant proteins in the medium was identified (Figure 2B) and purified (Figure 2C).

### 2.3. Biochemical Characterization of wtBmSUC1 and Mutants

**Figure 3:**
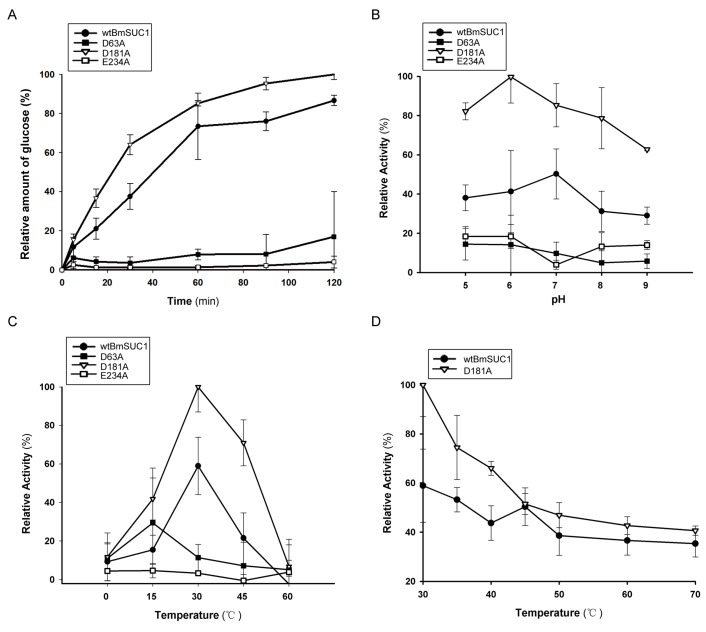
Comparison of enzymatic properties of wtBmSUC1 and mutants. (**A**) Influence of reaction time on hydrolysis activity; (**B**) Influence of pH on hydrolysis activity; (**C**) Influence of reaction temperature on hydrolysis activity; (**D**) Thermostability profiles of wtBmSUC1 and D181A. The hydrolysis activity was measured in 200 μL of culture mixture using sucrose as the substrate. Vertical bars indicate the mean ± SD (*n* = 3).

Sucrose was used as substrate to detect enzymatic characteristics of wtBmSUC1 and the three mutants. The results demonstrated that, as reaction time increased, both wtBmSUC1 and D181A increased glucose production, while the D63A and E234A mutants displayed essentially no enzyme activity at any time point (Figure 3A). Surprisingly, the specific activity of D181A was consistently higher than that of wtBmSUC1 (Figure 3A). We investigated the biochemical properties of the enzymes, including active pH range and optimal temperatures. D181A showed maximum activity at the same temperature (30 °C) as wtBmSUC1 (Figure 3C), while it had broader and higher activity than wtBmSUC1 from pH 5 to 8 (Figure 3B). For D63A and E234A, the activity was reduced to an extremely low level in all of the conditions, suggesting that these two sites are key enzyme catalytic residues (Figure 3A–C). Additionally, examination of thermo stability indicated that D181A activity rapidly decreased with increased temperature and 50% loss of activity (T_50_) for wtBmSUC1 and D181A was found at 70 °C and 45 °C, respectively (Figure 3D), indicating that the thermo stability of D181A was inferior to that of wtBmSUC1 in this experiment.

### 2.4. Comparison of Substrate Affinity and Catalytic Efficiency between wtBmSUC1 and D181A

β-FFase is able to liberate reducing sugars from fructosyl-β (2→1)-linked carbohydrates, such as sucrose and raffinose. We compared the substrate affinity and catalytic efficiency to sucrose and raffinose by calculating *K*_m_, *K*_cat_ and the *K*_cat_/*K*_m_ ratio based on the double reciprocal plots. A comparison of the *K_m_* values showed that both wtBmSUC1 and D181A had higher affinity to raffinose than to sucrose. The mutation of D181A enhanced its affinity to these two substrates, suggesting D181A had an improved substrate specificity when compared to wtBmSUC1, especially to the trisaccharide raffinose (Table 3, Figure S2). The *K*_cat_/*K*_m_ ratios indicated that both wtBmSUC1 and D181A could hydrolyze raffinose more efficiently than sucrose and D181A had about two times greater catalytic efficiency on the two substrates than wtBmSUC1 (Table 3, Figure S2). We concluded that the mutation of D181A promoted the enzymatic activity and kinetic properties of BmSUC1 (Figure 3, Table 3, Figure S2).

### 2.5. Quantitative Determination of Transfructosylating Activity

**Figure 4:**
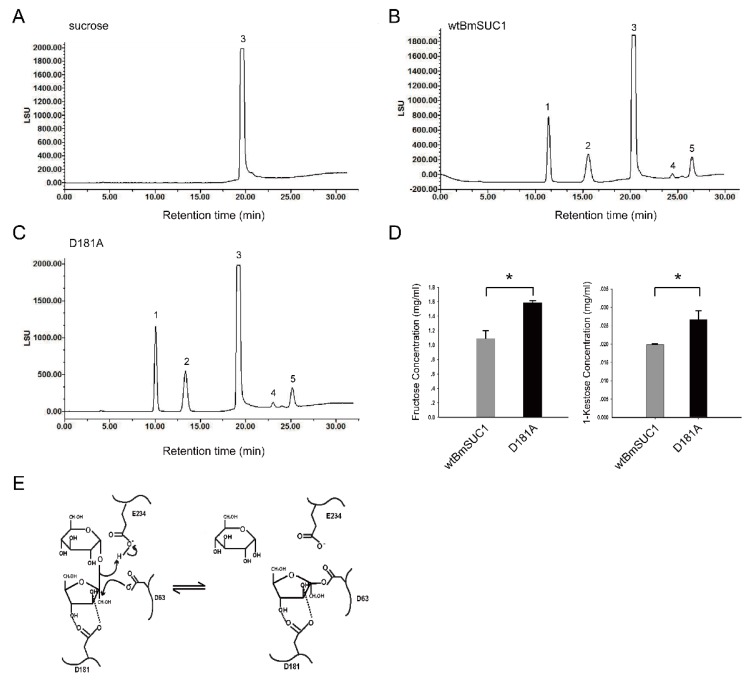
Determination of transfructosylation activity using sucrose as substrate and quantitative analysis on wtBmSUC1 and D181A by HPLC-ELSD (**A**–**D**). (**A**) Sucrose control without addition of enzyme in the reaction mixture; Peaks in (**B**,**C**) indicate fructose (1), glucose (2), sucrose (3), 1-kestose (4) and 6-kestose (5), respectively; (**D**) Comparison of fructose and 1-kestose catalyzed by wtBmSUC1 and D181A. * *p* < 0.05; (**E**) possible reaction mechanism of BmSUC1 acting as β-FFase. D63, D181 and E234 represent the nucleophile, transition-state stabilizer, and acid/base catalyst, respectively [25].

β-FFase can transfer fructose to the sucrose skeleton to synthesize short-chain FOS, such as 1-kestose, 6-kestose and others. Therefore, to determine if wtBmSUC1 and D181A have transfructosylating activity and if D181A is enhanced, we performed an enzymatic assay by using a high concentration of sucrose in the reaction mixture and prolonging the reaction time to 30 min. The reaction products were separated and quantitatively examined by a HPLC-Evaporative Light Scattering Detector (HPLC-ELSD) system (Figure 4). Retention time of standard fructose, glucose, sucrose, and 1-kestose were first tested. The sucrose mixture without the addition of enzyme was used as a blank control (Figure 4A). Analysis of the reaction products confirmed that both wtBmSUC1 and D181A were able to hydrolyze sucrose to release glucose and fructose. They were also able to catalyze the synthesis of 1-kestose (peak 4) and 6-kestose (peak 5, Figure 4B,C). The amounts of fructose produced by D181A and wtBmSUC1 were 1.587 and 1.089 mg/mL (Figure 4D, left) and 1-kestose were 0.027 and 0.019 mg/mL (Figure 4D, right), respectively. These data supported the hypothesis that both hydrolyzing and transfructosylating activities of D181A were significantly (*p* < 0.05) enhanced when compared to those of wtBmSUC1.

## 3. Discussion

Cloning and identification of *BmSuc1* from *B. mori* provided the first molecular evidence that β-FFase genes exist in the animal genomes [16]. BmSUC1 is a novel β-FFase in *B. mori* and might play an important role to protect against mulberry alkaloid damage for sucrose absorption [16]. Although homologous genes of *BmSuc1* have been found in other insects [18,19,20,21], information about the catalytic characters and enzymatic core sites for such insect β-FFases is limited.

The Bac-to-Bac baculovirus expression system is a novel gene expression system that allows the rapid and efficient generation of exogenous gene [26,27]. When an exogenous gene is cloned into the pFastBac vector and transformed into DH10Bac competent cells which includs bacmid, the mini-Tn7 component of pFastBac vector could transport the exogenous gene to the bacmid mini-Tn7 position. Therefore, this recombinant bacmid could transfect into the cell and over express the exogenous protein [26]. To understand the relative enzyme activity parameter of BmSUC1, we over-expressed the wtBmSUC1 and three mutants in BmN cells using a Bac-to-Bac/BmNPV expression system (Figure S1) [27]. The result that the majority of recombinant proteins were purified from the culture medium, not from the intracellular portion (Figure 2), supported that BmSUC1 is a secretory protein with an apparent signal peptide at the amino terminus (http://www.cbs.dtu.dk/services/SignalP/). This supports BmSUC1 acting as a sucrase in the larval gut lumen after being expressed in the goblet cells and secreted outside [16]. At 72 h post infection with the P2 virus resulted in the most BmN cells being broken and intracellular BmSUC1 flowing into the medium.

β-FFase catalyzes both the release of β-fructose from β-d-fructofurnoaside containing substrates, such as sucrose and raffinose, and the synthesis of short-chain FOS by transferring fructosyl moieties to link to the sucrose [1,2,3]. β-FFase belongs to the large glycoside hydrolase family GH32, in which three core motifs (NDPNG, RDP, and EC), each including an important acidic residue, have been identified as responsible for substrate binding and catalytic reaction [23] (Figure 1). Corresponding residues in BmSUC1 are Asp63, Asp181, and Glu234, which are highly conserved in β-FFases from originally different organisms (Figure 1). Therefore, we produced three mutant BmSUC1, D63A, D181A, and E234A, by site-directed mutagenesis (Figure S1A) and purified them for comparative enzymatic analysis (Figure 2C).

For the core active sites of β-FFase, crystal structure analysis indicated that the three carboxylate groups point to the center of the depression and generate a high negative charge at the active site [28]. Early studies on the yeast extracellular invertase identified Asp23 (NDPNG-motif, Asp63 in BmSUC1) as the nucleophile and Glu204 (EC-motif, Glu234 in BmSUC1) as the acid/base catalyst [29] (Figure 4E). These are located at appropriate distances and orientations from the substrates for their proposed catalytic roles in the active site [25]. Our data indicated that both D63A and E234A displayed little activity, verifying that the two sites are key amino acids for BmSUC1 (Figure 3A–C). The result is consistent with studies on Bacillus subtilis and other GH32 members [30,31]. In the double displacement mechanism, the acid/base catalyst acts as a general acid donating a proton to the glycosyl leaving group [4]. In the second step, the acid/base catalyst acts as a general base, removing a proton from the incoming fructosyl acceptor, which hydrolyses the fructose-enzyme intermediate [31,32]. These findings suggest that Asp63 probably works as the nucleophile and Glu234 as the acid/base catalyst in BmSUC1 (Figure 4E). When Asp63 and Glu234 are replaced by Ala, they lost the carboxyl so that they were unable to deliver protons. As a result, D63A and E234A showed almost no enzymatic activity (Figure 3A–C).

The other aspartate (RDP-motif, Asp181 in BmSUC1) seems not to be directly involved in the catalytic mechanism and probably acts as a transition-state stabilizer [25,30]. This residue forms hydrogen bonds to O3 and O4 of the fructose unit, whereas the neighboring Arg is hydrogen-bonded to the glucose O4 [25,28] (Figure 4E). The Asp mutant D191A of *Aspergillus japonicas* completely lost activity [25] and that of *Arthrobacter globiformis* did not show an obvious decrease in activity [33]. In *B. mori*, the D181A mutation markedly enhanced BmSUC1 activity in both hydrolysis and transfructosylation (Figure 3 and Figure 4). These findings indicate that this Asp residue of β-FFases in various species might interact with different neighboring residues to form hydrogen bonds that can exert an effect on stability and exercise different functions. Whether the D181A mutation increases the stability of the transition-state substrate can be further examined by constructing a three dimensional structure model of D181A/substrate complex.

Hydrophobic forces, stacking interactions, and hydrogen bonds were proposed as the dominant interactions in general protein-carbohydrate complexes [34,35]. The active site of BmSUC1 also contains several conserved hydrophobic residues that provide hydrophobic interactions with substrates, in which Pro82 interacts with the fructosyl unit of sucrose [25,36]. Previous mutagenesis studies at the WMNDPN motif region revealed its effects on substrate specificity and the type of catalytic reaction. When the Asn and Trp residues were replaced by hydrophilic residues, the transferred products were changed, indicating that the Asn and Trp residues determine product specificity [37,38,39]. The access of water to the general acid/base catalyst (Glu234 in BmSUC1) to receive the bound fructosyl group is therefore much restricted, which might be the reason that the main function of β-FFase is as a transferase or hydrolase [25] (Figure 4E).

Our results were similar to a study on *Schwanniomyces occidentalis* [3]*,* in which BmSUC1 synthesized 1-kestose and 6-kestose and 6-kestose is the main FOS product (Figure 4B). This suggests that BmSUC1 can split off a fructosyl residue from a substrate and retain this residue in the active site while releasing the substrate; afterwards, the fructosyl residue is released to water in the case of invertase and transferred to a FOS-chain in the case of fructosylferase [40,41] (Figure 4E). However, neokestose is the main transglycosylating product in *Xanthophyllomyces dendrorhous* [42], which can also synthesize 1-kestose and tetrasaccharides [1]. FOS synthesized by the plant vacuolar invertase is the main 1-kestose [40]. It is clear that fructosyltransferases catalyze the production of fructans from sucrose and the type of FOS depends on the kind of fructan synthesized by the organism. When a fructosyl-enzyme intermediate is formed, an increased concentration of the substrate sucrose can increase the proportion of fructosyltransferase activity [37,43]. Our finding that a D181A mutation significantly increased the fructosyltransferase activity of BmSUC1 (Figure 4C,D) increases the potential for using such a mutant to produce, in vitro, FOS that may be beneficial to human health.

β-FFases of plants, fungi and bacteria have been previously reported. As a recently discovered animal type β-FFase member, the amino acid sequence of BmSUC1 is most highly conserved to bacterial and insect β-FFases (Figure 1, Table 1, [16]). In the phylogenetic analysis of *A. planipennis*, the clustering of insect and bacterial β-FFases was supported with a high bootstrap value, indicating bacteria-to-insect HGT of these genes [21]. Based on studies about substrate affinity, many bacterial β-FFases have shown greater affinity to oligosaccharides, such as raffinose, 1-kestose, nystose than to sucrose [44,45,46]. The result that both wtBmSUC1 and the D181A mutant displayed much better affinity to raffinose than to sucrose (Table 3, Figure S2), provides support for the HGT of bacterial β-FFase genes to insects. HGT is a common feature of insect genomes and it promotes the ability of insects to adapt to novel environments. This study provides a better understanding of the biochemical properties of insect fructosyltransferases and their function in the FOS biosynthesis pathway. The biological significance of BmSUC1 for *B. mori* development and adaptation to the mulberry defense system remains unclear and it is worthy of advanced study.

## 4. Materials and Methods

### 4.1. Plasmid and Cell Strains

*Escherichia coli* DH5α and BL21 (DE3) strains were purchased from TransGen Biotech Co., Ltd. (Beijing, China). The Bac-to-Bac expression system including *Bombyx mori* nucleopolyhedrovirus (BmNPV) bacmid-containing *E. coli* strain DH10Bac and the pFastBac^TM^ Dual vector plasmid, pMD19T-GFP, and pET24b were maintained in our laboratory. The silkworm ovary derived cell line BmN was kindly provided by Dr. Anjiang Tan at Shanghai Institute of Plant Physiology and Ecology, Chinese Academy of Sciences and cultured in TC-100 insect medium (Ximeijie, Beijing, China) with 10% fetal bovine serum (ExCellbio, Shanghai, China) and 1% penicillin-streptomycin (Hyclone, Logan City, UT, USA) at 27 °C.

### 4.2. Sequence Analysis

The alignment of multiple acid amino sequences was conducted using BmSUC1 and β-FFases from fungi, bacteria, plants, and several species of Lepidoptera and Coleoptera by using software DNAMAN V6 (Lynnon, San Ramon, CA, USA). All of the β-FFase amino acid sequences can be obtained from GenBank (http://www.ncbi.nlm.nih.gov).

### 4.3. Over-Expression of BmSuc1 in E. coli and Preparation of Polyclonal Antibody

To construct a prokaryotic expression vector, the coding region of *BmSuc1* with 6xHis-tag sequence at the C-terminus was amplified by PCR from a silkworm larva midgut cDNA template, which was maintained in our lab. The oligonucleotide primers used in PCR are listed in Table 2. Following, the PCR product was connected with the pMD19T (TaKaRa, Dalian, China), according to the instructions for sequencing verification. After verification, the PCR product was digested with *Hind* III and *Not* I and ligated into the pET24b vector by 16 °C 2 h within T4 ligase, and then the prokaryotic expression plasmid pET24b-BmSuc1 with 6xHis-tag was transformed into *E. coli* BL21 (DE3) competent cells. Expression and purification of recombinant BmSUC1 was performed as described by Rao et al. [47]. 

About 3 mg of purified recombinant protein was collected for the preparation of the rabbit polyclonal anti-BmSUC1 antiserum, as described by Wang et al. [48]. The antiserum was produced by the Huaan biotechnology company using the purified recombinant BmSUC1 protein (Hangzhou, China). Total four times of subcutaneous immuno-injection were performed at multiple points of a New Zealand rabbit. For the first time, 0.5 mL purified BmSUC1 (1 mg/mL) was sufficiently mixed with equal volume of complete adjuvant and injected. Two weeks later, the same volume of protein (0.5 mg/mL) with equal volume of incomplete adjuvant was injected and repeated for another two times by 7 d interval each. After the third injection, arterial serum was sampled to check the quality by indirect ELISA using goat anti-rabbit HRP as antibody. At the end, whole blood was collected and purified by affinity chromatography column. Anti-BmSUC1 antiserum was eluted with 0.2 M glycine (pH 7.0) by a real-time protein monitor DH-4 (Huxi, Shanghai, China), regulated to pH 7.0 with Na_2_HPO_4_/NH_2_PO_4_ solution (1 M) and stored at −80 °C for use.

### 4.4. Site-Directed Mutagenesis of BmSUC1

Four pairs of primers were designed to amplify the overlap extension PCR products containing a site mutagenesis of Asp63Ala (D63A), Asp181Ala (D181A), or Glu234Ala (E234A) (Table 2) (Invitrogen, Shanghai, China). To produce each mutant, three PCR runs were carried out. Primers BmSUC1F and M1 were used for the first, M2 and BmSUC1R for the second reaction, and pMD19T-BmSuc1 was used as a template. For the third PCR BmSUC1F and BmSUC1R were used as primers with the mixed DNA products from above two reactions as template. Also, the primer pair BmSUC1F/R was used to amplify the normal coding region of *BmSuc1* from pMD19T-BmSuc1. Primer pair GFPF/R was used to clone the report gene GFP from pMD19T-GFP (Table 2). PrimeStar HS DNA polymerase (TaKaRa, Dalian, China) was used in the PCR reactions. A 6xHis tag sequence was added to each protein at the C terminus (Table 2).

### 4.5. Construction and Identification of Recombinant Bacmids

PCR products were purified using a DNA purification kit (Promega, Madison, WI, USA), digested with *Not* I and *Hind* III (TaKaRa, Dalian, China), cloned into the multiple cloning site with polh promoter of the pFastBac^TM^ Dual vector and confirmed by sequencing (Invitrogen, Shanghai, China). The GFP gene was cloned into the other multiple cloning site (*X-hol* I and *Kpn* I) with p10 promoter of the pFastBac^TM^ Dual vector. Recombinant plasmids were extracted by SanPrep Column Plasmid Mini-Preps Kit (Sangon, Shanghai, China) and transformed into *E. coli* DH10Bac (BmNPV) competent cells. Recombinant bacmid was extracted by the routine alkali lysis method. The upstream and downstream M13 common primers were used to identify positive recombinant bacmids.

### 4.6. Expression in BmN Cells and Purification of Recombinant Proteins

Recombinant bacmids were transfected into BmN cells using Lipofectamine2000 Reagent, following manufacturer instructions (Lifetechnology, Shanghai, China). After 3 d of culture at 27 °C, the cell cultures were collected and centrifuged at 1000× *g* for 5 min to remove cells and debris and to obtain the supernatant passage 0 virus (P0). P0 virus was used to re-infect the cells to obtain a higher titer of virus solution in P1 and then P2. The viral titer was determined according to the expression level of GFP using a fluorescent microscope Olympus DP72 (Olympus, Tokyo, Japan).

Three days after P2 bacmids infection, 250 mL of BmN cell cultures were centrifuged at 4000 rpm, 4 °C for 40 min to separate the medium and cells. The cells were disrupted in a lysis buffer (50 mM NaH_2_PO_4_, 300 mM NaCl, 10 mM imidazole, pH = 8.0) on ice using an ultrasonic cell disruptor for 10 min. The supernatant and pellet were separated by centrifuging at 12,000 rpm, 4 °C for 10 min. The pellet was resuspended in lysis buffer and in 8 M urea. To purify the recombinant wild type and mutant proteins from the culture medium, a Ni-NTA agarose (QIAGEN GmbH, Hilden, German) column was established and washed with an elution buffer (50 mM NaH_2_PO_4_, 300 mM NaCl, 250 mM imidazole, pH = 8.0) and was balanced with lysis buffer. The proteins in the medium were filtrated through filter paper and incubated in the Ni-NTA agarose column at 4 °C for 40 min. After the remanent proteins drained away from Ni-NTA agarose column, the protein was eluted with lysis buffer and concentrated, as described by Daimon et al. [16]. The purified protein was then added to Tris-HCl buffer (pH 7.4), according to a previous study [49], and stored at 4 °C or −80 °C until use.

### 4.7. SDS-PAGE and Western Blot

Expression products in the medium, cell supernatant and pellet, purified proteins were determined by 10% sodium dodecylsulfate–polyacrylamide gel electrophoresis (SDS-PAGE). The fresh medium was used as a negative control. After SDS-PAGE, the proteins were then transferred to a polyvinylidene fluoride (PVDF) membrane (Millipore, Billerica, MA, USA) using Trans-Blot (Bio-Rad, Hercules, CA, USA) at 20 V for 15 min. Immunoblot analysis was conducted, as described by Li, et al. [49]. Anti-BmSUC1 polyclonal antibody was used as the primary antibody (1:500) and goat anti-rabbit IgG as the secondary antibody (1:10,000) (Sangon, Shanghai, China). The final detection was performed by using Diaminobenzidine (DAB) Horseradish Peroxidase Color Development Kit (Sangon, Shanghai, China).

### 4.8. Enzymatic Activity Assay and Kinetic Analysis

Hydrolysis activity of BmSUC1 was assayed according to Daimon et al. [16] with some modifications. The standard 200 μL reaction mixture contained 1 μg of purified protein, 100 mM sucrose, and 10 mM Britton-Robinson buffer (pH 7.0). The reaction was initiated by the addition of the enzyme and was kept at 30 °C for 0–120 min and stopped by boiling for 5 min. Following, equal volumes of dinitrosalicylic acid (DNS) solution (44 mM 3,5-dinitrosalicylic acid, 400 mM NaOH, 1 M potassium sodium tartrate) were added to the reaction mixture and boiled for 5 min to measure the glucose liberated with the maximum UV absorbance at 540 nm. Reaction without enzyme addition was used as a control. One unit of enzyme was defined as the amount of the enzyme/μg of protein that catalyzed the production of 1 mmol of glucose/min (mmol/min/μg).

To study the effect of temperature on BmSUC1 activity, each reaction mixture was kept at temperatures ranging from 0 to 60 °C for 15 min. To study the effect of pH on BmSUC1 activity, the Britton-Robinson buffer (pH 5.0–9.0) was used. To estimate the thermostability of BmSUC1, 2 μg of purified protein was incubated at different temperatures (30–70 °C) for 10 min before substrate addition. Based on the double reciprocal plot (Lineweaver-Burk), kinetic parameters of Michaelis constant (*K*_m_), and catalytic rate constant (*K*_cat_) were determined using 2 μg of purified protein and sucrose concentrations ranging from 40 to 280 mM, and raffinose concentrations ranging from 10 to 70 mM.

### 4.9. HPLC-ELSD Analysis

To determine and compare both hydrolysis and transfructosylation activities between wtBmSUC1 and D181A mutant, 200 μL reaction mixture containing 2 μg of purified protein, 100 mM sucrose, and 10 mM Britton-Robinson buffer (pH 7.0) was kept at 30 °C for 30 min and boiled for 5 min to stop the reaction. After centrifugation at 3500× *g* for 5 min, the supernatant was filtered with a 0.22 μm membrane (Sangon, Shanghai, China) and subjected to high performance liquid chromatography (HPLC, Waters 600E) system (Waters, Millford, MA, USA).

The concentration of the different products was analyzed by HPLC with a quaternary pump (Waters600) coupled to a 5 μm Luna NH2 column (4.6 mm × 250 mm) (Phenomenex, Guangzhou, China). Detection was performed using ELSD (Waters 2424) equilibrated at 85°C. Acetonitrile: water 85:15 (*v*/*v*), degassed with helium, which was used as mobile phase at 0.9 mL min^−1^ for 8 min. Then, a gradient from this eluent to acetonitrile: water 75: 25 (*v*/*v*) was performed in 2 min, and held for 6 min. A new gradient to acetonitrile: water 70:30 (*v*/*v*) was performed in 5 min and held for 14 min. Total analysis time was 35 min. The column temperature was kept constant at 25 °C [4]. Chromatographic grade acetonitrile and the standard substances of fructose, glucose, sucrose, and 1-kestose were purchased from Aladdin (Shanghai, China). The data were analyzed by one-way analysis of variance followed by Dunnett’s test to localize the significant difference. A *p* value of less than 0.05 was considered statistically significant.

## Figures and Tables

**Table 1:** Identity of BmSUC1 with some β-FFases from other species.

Taxonomy	Species (Accession Number)	Identity (%)
Insect	*Helicoverpa armigera* (ABU98615)	43
	*Agrilus planipennis* (AIR93898)	40
	*Manduca sexta* (ACX49762)	39
	*Sphenophorus levis* (AIL92341)	39
Bacterium	*Bacillus licheniformis* (WP_003186470)	46
	*Bacillus megaterium* (KWU53447)	44
	*Staphylococcus pasteuri* (WP_023374434)	43
	*Thermotoga maritima* (1UYP_A)	33
Plant	*Arabidopsis thaliana* (AAA63802)	31
	*Cichorium intybus* (CAC37922)	30
Fungus	*Arthrobacter globiformis* (ADK46938)	31
	*Aspergillus japonicus* (WP_003803118)	13

**Table 2:** Oligonucleotide primers used in PCR.

Name of Primer Pair	Primer Sequence (5′→3′)
D63AM1/M2	GAAAAGCCGTTAGGGGCATTCATCCA
	TGGATGAATGCCCCTAACGGCTTT TC
D181AM1/M2	CCAAATCTTGGGGGCTCTGAAATCAGC
	GCTGATTTCAGAGCCCCCAAGATTTGG
E234AM1/M2	CATGCCCACATGTAGCCCAT
	ATGGGCTACATGTGGGCATG
BmSUC1F/R	ATTTGCGGCCGCTATGTTCGCCTGGAGC
	CCCAAGCTTTTA*ATGATGATGATGATGATG*AGCGGGTACACT
GFPF/R	CCGCTCGAGATGGTGAGCAAGGGC
	CGGGGTACCTTACTTGTACAGCTC

The underlined nucleotides indicate the position of altered codons. The red nucleotides indicate the restriction sites. Nucleotides in italic indicate the 6xHis tag.

**Table 3:** Kinetic parameters of BmSUC1 and D181A on different substrates.

Substrate	Enzyme	*K*_m_ (mM)	*K*_cat_ (S^−1^)	*K*_cat_/*K*_m_ (S^−1^mM^−1^)
Sucrose	BmSUC1	88.32 ± 11.75	0.022 ± 0.0009	2.49 × 10^−4^
	D181A	52.87 ± 6.85	0.025 ± 0.0004	4.73 × 10^−4^
Raffinose	BmSUC1	52.32 ± 17.22	0.025 ± 0.0028	2.91 × 10^−4^
	D181A	32.03 ± 4.43	0.021 ± 0.0014	6.56 × 10^−4^

*K*_cat_ values were calculated assuming a molecular mass of 56 kDa for the enzyme. The ± refers to standard errors based on the curve fitting using SigmaPlot 12.5 (SYSTAT, San Jose, CA, USA).

## Supplementary Materials

Supplementary materials can be found at http://www.mdpi.com/1422-0067/19/3/683/s1.

## Abbreviations

GH32	glycosyl-hydrolase family 32
β-FFase	β-fructofuranosidase
FOS	fructooligosaccharides
D-AB1	1,4-dideoxy-1,4-imino-d-arabinitol
DNJ	1-deoxynojirimycin
HGT	horizontal gene transfer
BmNPV	*Bombyx mori* nucleopolyhedrovirus
SDS-PAGE	sodium dodecylsulfate–polyacrylamide gel electrophoresis
PVDF	polyvinylidene fluoride
DNS	dinitrosalicylic acid
*K*_m_	kinetic parameters of Michaelis constant
*K*_cat_	catalytic rate constant
HPLC	high performance liquid chromatography
ELSD	evaporative light-scattering detector
T_50_	temperature of 50% loss of activity
