# Supplementary material for: D181A Site-Mutagenesis Enhances Both the Hydrolyzing and Transfructosylating Activities of BmSUC1, a Novel β-Fructofuranosidase in the Silkworm Bombyx mori
Source: Int J Mol Sci. 2018 Feb 28;19(3):683. doi: 10.3390/ijms19030683 (PMC5877544; doi:10.3390/ijms19030683)
Supplement: Supplementary file 1 [file ijms-19-00683-s001.pdf]

**Supplementary Materials:**

**Figure S1.** Site mutagenesis of BmSUC1 by overlapping extension PCR and Bac-to-Bac/BmNPV expression in BmN cells.

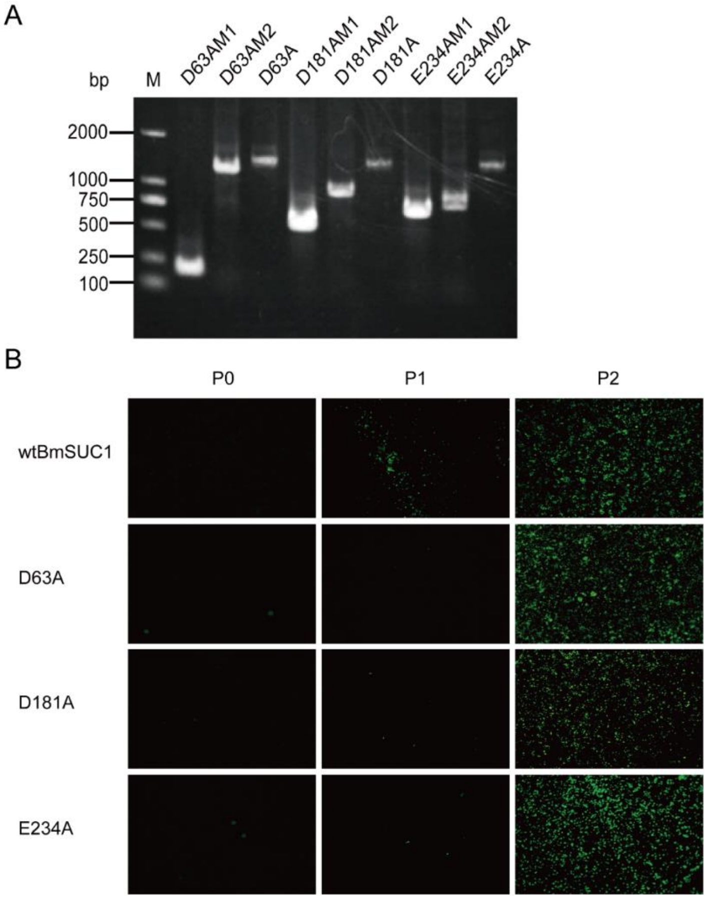


**Figure S1.** Site mutagenesis of BmSUC1 by overlapping extension PCR (**A**) and Bac-to-Bac/BmNPV expression in BmN cells (**B**). Each mutant was produced by three PCR reactions with primer pairs of BmSUC1F/M1, M2/BmSUC1R and BmSUC1F/R, respectively. The BmN cells were infected by recombinant bacmids to express wtBmSUC1, D63A, D181A and E234A. P0-P2, 3 d after infection by recombinant virus, passage 0 virus and passage 1 virus, respectively. GFP was used to present the expression of recombinant BmSUC1 visible under a fluorescence microscope (×100).

**Figure S2**. The enzymatic parameters of wtBmSUC1 and mutant D181A to substrates sucrose and raffinose.

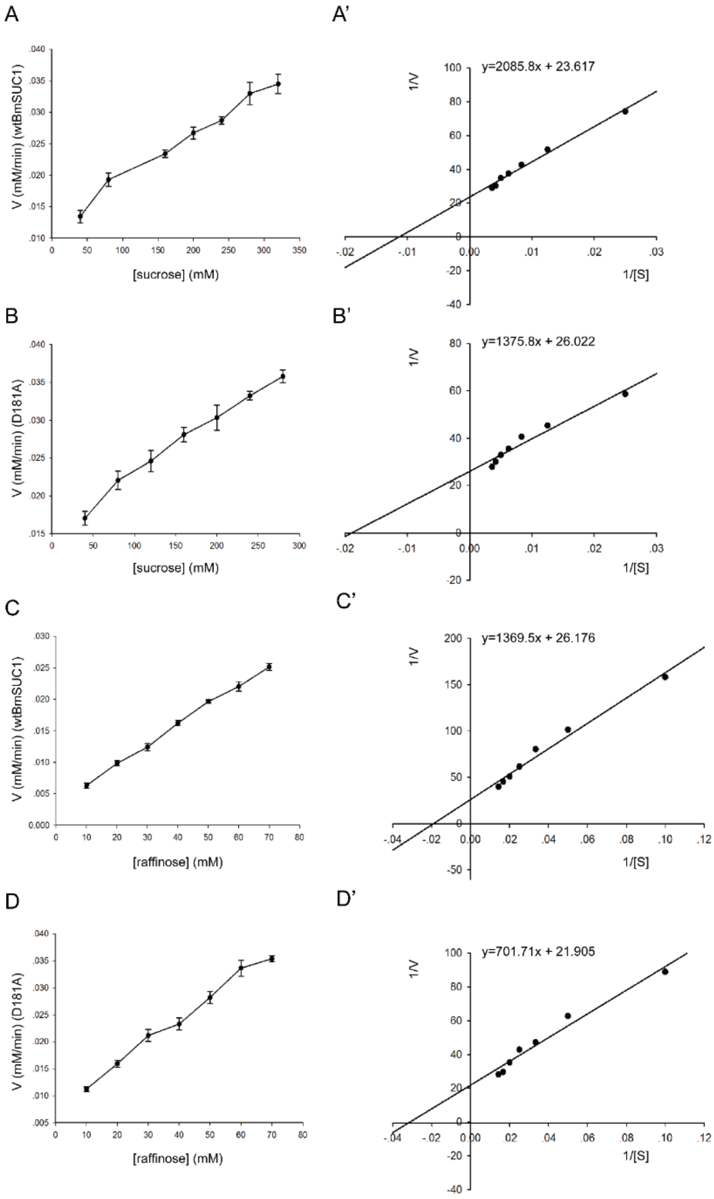


**Figure S2.** The enzymatic parameters of wtBmSUC1 and mutant D181A to substrates sucrose and raffinose. **A**, **B**, influence of sucrose concentration (40-280 mM) on the reaction rate. **A′**, **B′**, double reciprocal plot of reaction rate versus sucrose concentration. **C**, **D**, influence of raffinose concentration (10-70 mM) on the reaction rate. **C′**, **D′**, double reciprocal plot of reaction rate versus raffinose concentration. The reaction rate was calculated by the amount of glucose production from the reaction within 15 min. Reaction without the addition of the enzyme was used as a control. The bars indicate the mean ± SD (n=3).
